# An Exact Solution for Power-Law Fluids in a Slit Microchannel with Different Zeta Potentials under Electroosmotic Forces

**DOI:** 10.3390/mi9100504

**Published:** 2018-10-05

**Authors:** Du-Soon Choi, Sungchan Yun, WooSeok Choi

**Affiliations:** Department of Mechanical Engineering, Korea National University of Transportation, Chungju, 27469, Korea; dschoi@ut.ac.kr

**Keywords:** Electroosmosis, Power-law fluid, Non-Newtonian fluid, Asymmetric zeta potential

## Abstract

Electroosmotic flow (EOF) is one of the most important techniques in a microfluidic system. Many microfluidic devices are made from a combination of different materials, and thus asymmetric electrochemical boundary conditions should be applied for the reasonable analysis of the EOF. In this study, the EOF of power-law fluids in a slit microchannel with different zeta potentials at the top and bottom walls are studied analytically. The flow is assumed to be steady, fully developed, and unidirectional with no applied pressure. The continuity equation, the Cauchy momentum equation, and the linearized Poisson-Boltzmann equation are solved for the velocity field. The exact solutions of the velocity distribution are obtained in terms of the Appell’s first hypergeometric functions. The velocity distributions are investigated and discussed as a function of the fluid behavior index, Debye length, and the difference in the zeta potential between the top and bottom.

## 1. Introduction

Recently, microfluidic device applications are increasing in the fields of chemical analysis, medical diagnostics, material synthesis, and others [1,2,3]. In the field of microfluidics, flow control in a microchannel is one of the most important issues. The problem with conventional pressure-driven flow is that as the channel size decreases, the hydraulic area becomes extremely small, resulting in a significant increase in the corresponding hydraulic resistance [4]. Electroosmotic flow (EOF) does not suffer from this problem because it is the motion of fluid that depends on the electric field across a microchannel [5,6,7].

Many efforts have been made to study electroosmotic flow (EOF) using Newtonian fluids. However, a few microfluidic devices are used more frequently for processing biological fluids such as blood, saliva, DNA, and polymer solutions, which cannot be treated as Newtonian fluids. To analyze the EOF of such fluids, an approach to non-Newtonian constitutive relations should be considered [8,9,10,11,12,13,14,15].

Among the various constitutive laws for non-Newtonian fluids, the power law model is the most popular because of its simplicity and suitability for analyzing a wide range of fluids. Thus, many researchers have conducted EOF studies using the power law model [16,17,18,19,20,21,22,23,24,25]. Zhao et al. analyzed the EOF of power-law fluids and obtained the approximate solution of the velocity field in a slit microchannel [16]. In addition, they studied a general Smoluchowski slip velocity over a surface [17] and provided an exact solution of the velocity distribution in a slit microchannel [18]. They also analyzed the EOF of power-law fluids in cylindrical [19] and rectangular [20] microchannels. Tang et al. conducted a numerical study of the EOF in microchannels of a power-law fluid using the lattice Boltzmann method [21]. Vasu and De analyzed a mathematical model of the EOF of power-law fluids in a rectangular microchannel at high zeta potential [22]. Babaie et al. and Hadigol et al. numerically analyzed the EOF of power-law fluids in a slit microchannel with pressure gradient [23,24]. Ng and Qi developed a simplified analytical model to describe the electrokinetic flow of a power-law fluid for varying wall potentials and channel heights in a slit channel [25].

Most previous studies have been performed on microchannels with the same zeta potential at the top and bottom walls. To our knowledge, however, many microfluidic devices are made with a combination of different materials, such as silicon dioxide (glass) as the base and polydimethylsiloxane as the top and side-walls. In these cases, asymmetric electrochemical boundary conditions should be applied for reasonable analysis of the EOF. Afonso et al. [26] and Choi et al. [27] analyzed the EOF of viscoelastic fluids in a microchannel with asymmetric zeta potential using the simplified Phan–Thien–Tannar model. Qi and Ng investigated the EOF of a power-law fluid through a slit channel where the walls were asymmetrically patterned with periodic variations in shape and zeta potential [28]. Hadigol et al. numerically investigated the characteristics of electroosmotic micromixing of power-law fluid in a slit microchannel with nonuniform zeta potential distributions along the walls of the channel [29]. Jiménez et al. investigated the start-up from rest of the EOF of Maxwell fluids in a rectangular microchannel with asymmetric high zeta potentials at the walls [30]. Peralta et al. conducted theoretical analysis of the start-up of oscillatory EOF in a parallel-plate microchannel under asymmetric zeta potentials [31]. Recently, Choi et al. presented the EOF in a rectangular microchannel using numerical analysis [32] and suggested an approximate solution for the EOF of power-law fluid with asymmetric zeta potential of a planar channel [33].

Obtaining the exact solution not only provides physical insight into the phenomena but can also serve as a benchmark for experimental, numerical, and asymptotic analyses. Zhao and Yang [18] have reported the exact solution for the EOF of a power-law fluid with a symmetric zeta potential. However, it remains a challenge to obtain the exact solution of the EOF in microchannels with asymmetric electrochemical boundary conditions.

In the present study, exact solutions for EOFs of power-law fluids in a slit microchannel with different zeta potentials at the top and bottom walls are presented. In addition, the key parameters affecting the velocity distribution of EOF, including the fluid behavior index, Debye length, and different zeta potentials at the top and bottom walls are analyzed.

## 2. Mathematical Formulation

**Figure 1:**
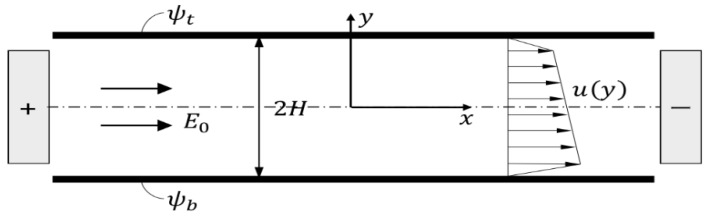
Schematic diagram of electroosmotic flow in a slit microchannel.

Figure 1 shows a two-dimensional EOF in a slit microchannel of height 2*H*. The top and bottom walls were charged with zeta potential *ψ_t_* and *ψ_b_*, respectively. An external electric field *E*_0_ was applied to a power-law fluid with a constant density *ρ* and electric permittivity *ϵ*.

The velocity field in the microchannel is governed by the continuity and Cauchy momentum equations given as:

(1) ∇⋅v=0,

(2) $$\rho \frac{Dv}{Dt}=-\nabla p+\nabla \cdot \tau +F,$$

where **v** = (*u*, *v*) is the velocity vector, *p* is the pressure, ***τ*** is the stress tensor, and **F** = (*F_x_*, *F_y_*) is the body force. The stress tensor can be given by

(3) $$\tau =\mu \left(\nabla v+\nabla v^{T}\right),$$

where *μ* is the effective viscosity.

For a steady, fully developed, unidirectional flow with no applied pressure and negligible gravitational force, the body force acts only along the *x*-direction and the Cauchy momentum equation in Equation (2) can be simplified as:

(4) $$\frac{d}{dy}\left(\mu \frac{du}{dy}\right)+F_{x}=0.$$

The effective viscosity of the power-law fluid can be expressed as:

(5) $$\mu =m{\left|\frac{du}{dy}\right|}^{n-1},$$

where *m* is the flow consistency index, and *n* is the flow behavior index.

The body force along the *x*-direction is given by:

(6) $$F_{x}=\rho_{e}E_{0}.$$

The net charge density *ρ_e_* can be obtained by the Poisson equation, which takes the form of:

(7) $$\varepsilon \frac{d^{2}\psi }{dy^{2}}=-\rho_{e}.$$

With the assumption of Boltzmann distribution and small zeta potentials, the electrical potential profile in the electrical double layer (EDL) is governed by the linearized Poisson–Boltzmann equation expressed by:

(8) $$\frac{d^{2}\psi }{dy^{2}}=\kappa^{2}\psi ,$$

which is subject to the following boundary conditions:

(9) $$\psi {|}_{y=H}=\psi_{t},\;\psi {|}_{y=-H}=\psi_{b}.$$

*κ*^−1^ is called the Debye length and is defined as *κ*^−1^ = (*εk*_B_*T*/2*e*^2^*z*^2^*n*_∞_)^1/2^, where *n*_∞_ and *z* are the bulk number concentration and the valence of ions, respectively, *e* is the fundamental charge, *k*_B_ is the Boltzmann constant, and *T* is the absolute temperature.

The solution for the electrical potential distribution is of the form:

(10) $$\psi \left(y\right)=\frac{\psi_{t}+\psi_{b}}{2}\cdot \frac{\cosh\left(\kappa y\right)}{\cosh\left(\kappa H\right)}+\frac{\psi_{t}-\psi_{b}}{2}\cdot \frac{\sinh\left(\kappa y\right)}{\sinh\left(\kappa H\right)}.$$

Then, the net charge density *ρ_e_* can be expressed as a function of the EDL potential

(11) $$\rho_{e}\left(y\right)=-\kappa^{2}\varepsilon \psi \left(y\right).$$

With all the aforementioned considerations, the Cauchy momentum equation in Equation (4) is expressed as:

(12) $$\frac{d}{dy}\left[m{\left|\frac{du}{dy}\right|}^{n-1}\frac{du}{dy}\right]-\kappa^{2}\varepsilon E_{0}\psi \left(y\right)=0.$$

This equation is constrained by the following boundary conditions (no-slip conditions)

(13) $$u{|}_{y=-H}=0,\;u{|}_{y=H}=0.$$

Substituting Equation (10) into Equation (12) yields:

{(14a)ddy[(dudy)n]=κ2εE0m(ψt+ψb2⋅cosh(κy)cosh(κH)+ψt−ψb2⋅sinh(κy)sinh(κH)), if dudy≥0,(14b)ddy[(−dudy)n]=−κ2εE0m(ψt+ψb2⋅cosh(κy)cosh(κH)+ψt−ψb2⋅sinh(κy)sinh(κH)), if dudy<0.

Since most of the materials that make up the microchannels have negative zeta potential [34,35], the wall zeta potentials are assumed to be negative in the present study; thus, the flow occurs in the +*x*-direction (if *E*_0_ > 0). Let *y_c_* be the point where ${\frac{du}{dy}|}_{y=y_{c}}=0$, $\left(-H\leq y_{c}\leq H\right)$, then the velocity gradient is positive $\left(\frac{du}{dy}\geq 0\right)$ in the interval $-H\leq y\leq y_{c}$, and negative $\left(\frac{du}{dy}<0\right)$ in the interval $y_{c}\leq y\leq H$.

Integrating Equation (14) with *y* leads to:

dudy={(15a)(−κεE0ψmm)1n{−sinh(κy)cosh(κH)−R⋅cosh(κy)sinh(κH)+C+}1n, if −H≤y≤yc,(15b)−(−κεE0ψmm)1n{−(−sinh(κy)cosh(κH)−R⋅cosh(κy)sinh(κH)+C−)}1n, if yc<y≤H,

where *ψ_m_* and *R* are the average zeta potential and the dimensionless zeta potential difference between the top and bottom walls, respectively, which are defined by:

(16) $$\psi_{m}\equiv \frac{\psi_{t}+\psi_{b}}{2},$$

(17) $$R\equiv \frac{\psi_{t}-\psi_{b}}{\psi_{t}+\psi_{b}},$$

and *C*^+^ and *C*^−^ are integral constants.

Both Equations (15a) and (15b) should be zero at *y = y_c_*. Therefore,

(18) $$C^{+}=C^{-}=\frac{\sinh\left(\kappa y_{c}\right)}{\cosh\left(\kappa H\right)}+R\cdot \frac{\cosh\left(\kappa y_{c}\right)}{\sinh\left(\kappa H\right)}\equiv C.$$

Integrating Equation (15) with the corresponding boundary condition in Equation (13) leads to the velocity distribution:

u(y)={(19a)(−κεE0ψmm)1n∫−Hy{I(y′)+C}1ndy′, if −H≤y≤yc,(19b)−(−κεE0ψmm)1n∫Hy{−I(y′)−C}1ndy′, if yc<y≤H,

where

(20) $$I\left(y\right)\equiv -\frac{\sinh\left(\kappa y\right)}{\cosh\left(\kappa H\right)}-R\cdot \frac{\cosh\left(\kappa y\right)}{\sinh\left(\kappa H\right)},$$

By integrating Equation (19), the velocity distribution can be obtained as:

u(y)={(21a)us[V+(y)−V+(−H)], if −H≤y≤yc,(21b)us[−V−(y)+V−(H)], if yc<y≤H,

where *u_s_* denotes the generalized Smoluchowski velocity for power-law fluids by employing the average zeta potential *ψ_m_* at the top and bottom walls on the basis of the work of Zhao et al. [16], which is expressed as:

(22) $$u_{s}=n\kappa^{\frac{1}{n}-1}{\left(-\frac{\varepsilon E_{0}\psi_{m}}{m}\right)}^{\frac{1}{n}},$$

and

(23a) $$V^{+}\left(y\right)\equiv -\frac{1}{\left(n+1\right)\sqrt{C^{2}+w^{2}}}{\left[I\left(y\right)+C\right]}^{\frac{n+1}{n}}F_{1}\left(1+\frac{1}{n};\;\frac{1}{2},\;\frac{1}{2};\;2+\frac{1}{n};\;\frac{I\left(y\right)+C}{C+iw},\frac{I\left(y\right)+C}{C-iw}\right),$$

(23b) $$V^{-}\left(y\right)\equiv \frac{1}{\left(n+1\right)\sqrt{C^{2}+w^{2}}}{\left[-I\left(y\right)-C\right]}^{\frac{n+1}{n}}F_{1}\left(1+\frac{1}{n};\;\frac{1}{2},\;\frac{1}{2};\;2+\frac{1}{n};\;\frac{I\left(y\right)+C}{C+iw},\frac{I\left(y\right)+C}{C-iw}\right).$$

(24) $$y_{c}=\frac{1}{\kappa }\ln\left(\frac{C+\sqrt{C^{2}+w^{2}}}{\frac{R}{\sinh\left(\kappa H\right)}+\frac{1}{\cosh\left(\kappa H\right)}}\right).$$

(25) $$w=\sqrt{\frac{1}{{\cos h}^{2}\left(\kappa H\right)}-\frac{R^{2}}{{\sin h}^{2}\left(\kappa H\right)}}.$$

The integral constant *C* can be obtained from the following equation:

(26) J(C)=0,

where *J*(*x*) are defined by:

(27) $$J\left(x\right)={\left[I\left(-H\right)+x\right]}^{\frac{n+1}{n}}F_{1}\left(1+\frac{1}{n};\;\frac{1}{2},\;\frac{1}{2};\;2+\frac{1}{n};\;\frac{I\left(-H\right)+x}{x+iw},\frac{I\left(-H\right)+x}{x-iw}\right)\;-{\left[-I\left(H\right)-x\right]}^{\frac{n+1}{n}}F_{1}\left(1+\frac{1}{n};\;\frac{1}{2},\;\frac{1}{2};\;2+\frac{1}{n};\;\frac{I\left(H\right)+x}{x+iw},\frac{I\left(H\right)+x}{x-iw}\right).$$

The details of the mathematical derivations are described in Appendix A. It is a challenge to obtain the explicit form for the integral constant *C*. Thus, in this study, a numerical method was used for evaluating *C*.

*F*_1_(*a*; *b*_1_, *b*_2_; *c*; *x*, *y*) in Equation (23) is the Appell’s first hypergeometric function [36], which can be represented as a one-dimensional integral form [37]:

(28) $$\begin{array}{c} F_{1}\left(1+\frac{1}{n};\;\frac{1}{2},\;\frac{1}{2};\;2+\frac{1}{n};\frac{\gamma }{\alpha +i\beta },\;\frac{\gamma }{\alpha -i\beta }\right)=\frac{\Gamma \left(2+\frac{1}{n}\right)}{\Gamma \left(1+\frac{1}{n}\right)}\overset{1}{\underset{0}{\int }}\frac{t^{\frac{1}{n}}}{\sqrt{1-\frac{\gamma t}{\alpha -i\beta }}\sqrt{1-\frac{\gamma t}{\alpha +i\beta }}}dt\\ =\frac{n+1}{n}\overset{1}{\underset{0}{\int }}\frac{t^{\frac{1}{n}}}{\sqrt{\frac{\gamma^{2}t^{2}-2\alpha \gamma t+\left(\alpha^{2}+\beta^{2}\right)}{\alpha^{2}+\beta^{2}}}}dt, \end{array}$$

where *α*, *β*, and *γ* are real values, and *Γ*(*z*) is a gamma function. It is evident from Equation (28) that, although the Appell’s first hypergeometric function in Equation (23) has complex arguments, it always has a real value.

Alternatively, Equation (21) can be expressed in the single form using Equation (15) as follows:

(29) u(y)=V(y)−V(−H),

where

(30) $$V\left(y\right)\equiv -\frac{1}{\kappa }\frac{n}{n+1}\frac{1}{\sqrt{C^{2}+w^{2}}}\left(\frac{du}{dy}\right)\left[I\left(y\right)+C\right]F_{1}\left(1+\frac{1}{n};\;\frac{1}{2},\;\frac{1}{2};\;2+\frac{1}{n};\;\frac{I\left(y\right)+C}{C+iw},\frac{I\left(y\right)+C}{C-iw}\right).$$

Equation (21) is applicable to the EOF of power-law fluids with arbitrary zeta potentials at the top and bottom walls. If the top and bottom walls have the same zeta potential, then the velocity distribution is expressed as follows:

(31) $$u\left(y\right)=u_{s}\left[V_{symm}\left(H\right)-V_{symm}\left(y\right)\right],$$

where

(32) $$V_{symm}\left(y\right)\equiv \frac{{\left(-1\right)}^{\frac{n-1}{2n}}}{n}\frac{\cosh\left(\kappa y\right)}{\cosh^{\frac{1}{n}}\left(\kappa H\right)}{}_{2}F_{1}\left(\frac{1}{2},\;\frac{n-1}{2n};\;\frac{3}{2};\;{\cos h}^{2}\left(\kappa y\right)\right)$$

which is identical to the result of Zhao and Yang [18] on the EOF of power-law fluid with a symmetrical zeta potential. The detailed derivations are described in Appendix B.

## 3. Results and Discussions

The key parameters that affect velocity distribution are the fluid behavior index *n*, electrokinetic parameter *κH*, and dimensionless zeta potential difference *R* = (*ψ_t_* − *ψ_b_*)/(*ψ_t_* + *ψ_b_*) between *ψ_t_* and *ψ_b_*. In this section, the effects of these parameters on velocity distribution are investigated.

**Figure 2:**
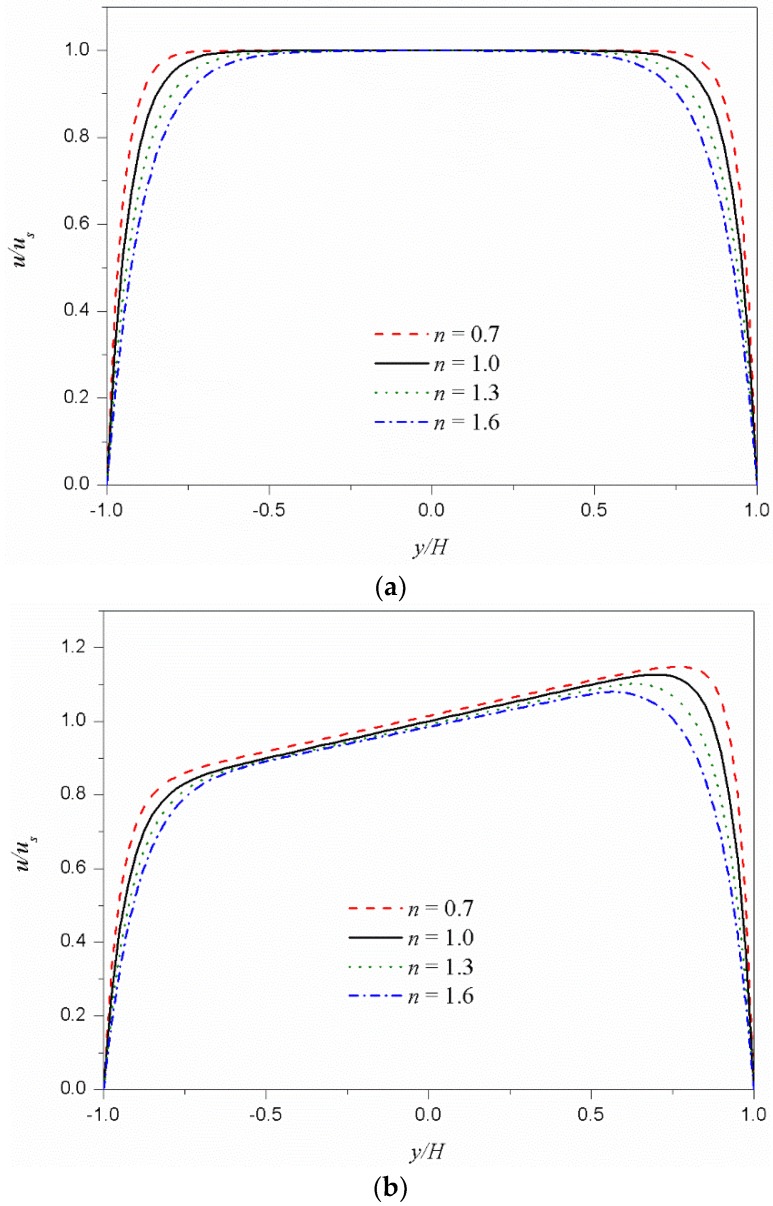
Dimensionless velocity distributions *u*/*u_s_* for different values of the fluid behavior index *n* under *κH* = 15. (**a**) Symmetric zeta potentials (*ψ_t_*/*ψ_b_* = 1). (**b**) Asymmetric zeta potentials (*ψ_t_*/*ψ_b_* = 1.5).

Figure 2 shows the dimensionless velocity (*u*/*u_s_*) distributions from Equation (21) for different values of fluid behavior index *n* at a fixed *κH* of 15. Figure 2a represents the velocity distributions with same zeta potentials (*R* = 0) at the bottom and top, while Figure 2b indicates those of asymmetric zeta potentials with *R* of 0.2 (*ψ_t_*/*ψ_b_* = 1.5). In both cases of symmetric and asymmetric zeta potentials, as the fluid behavior index *n* decreases, the velocity gradient near the wall increases, and the plug-like characteristics of velocity distribution are enhanced. This is because the fluid with smaller fluid behavior index is less viscous, and the velocity can easily change from zero at the wall to the Smoluchowski velocity at the core region.

**Figure 3:**
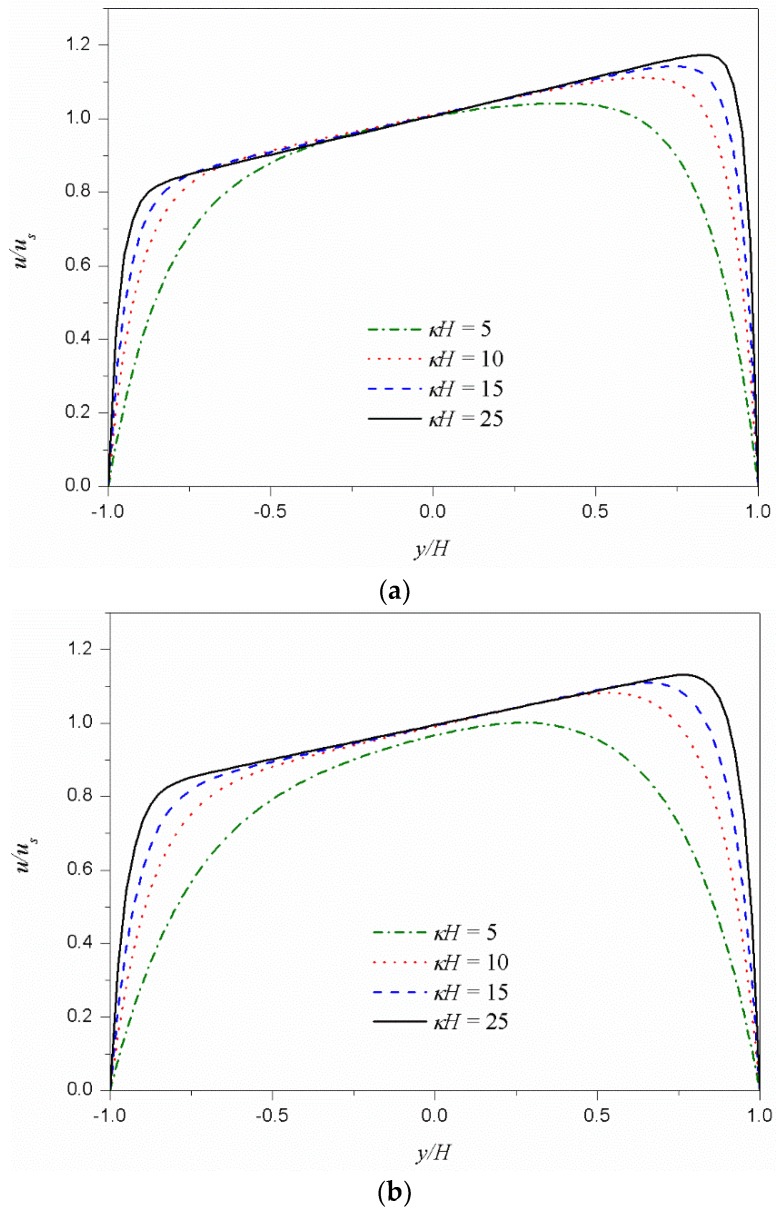
Dimensionless velocity distributions *u*/*u_s_* for different values of *κH* under *ψ_t_*/*ψ_b_* = 1.5. (**a**) Shear thinning fluid (*n* = 0.8). (**b**) Shear thickening fluid (*n* = 1.2).

Figure 3 shows the dimensionless velocity (*u*/*u_s_*) distributions for different values of *κH*. Figure 3a shows the velocity distributions of the shear thinning fluid (*n* = 0.8) and Figure 3b shows those of the shear thickening fluid (*n* = 1.2). In both cases, as *κH* increases, the velocity distribution changes from parabolic type to plug-like type. The increase of *κH* means a decrease in Debye length. In other words, the EDL thickness, on which the electrostatic body force is applied, decreases and the velocity distribution changes to a plug-like type.

**Figure 4:**
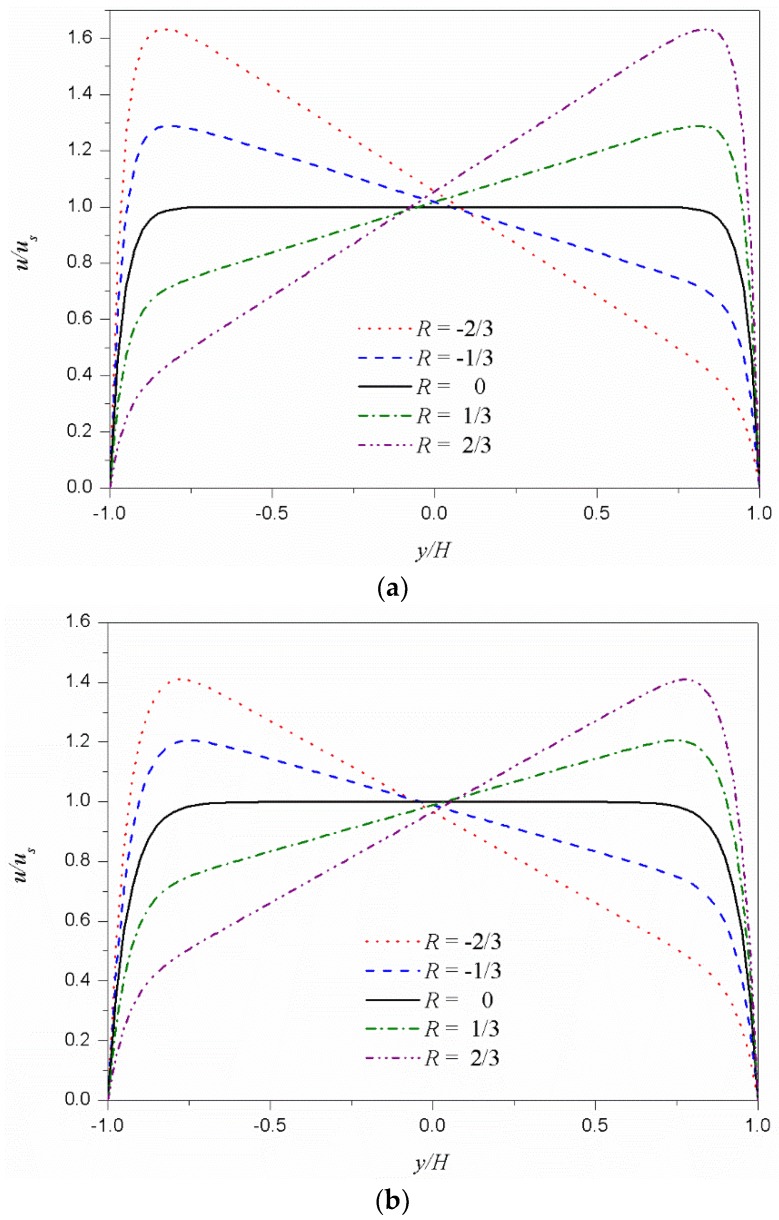
Dimensionless velocity distributions *u*/*u_s_* as a function of the dimensionless zeta potential difference *R.* The values −2/3, −1/3, 0, 1/3, and 2/3 of *R*, correspond to 0.2, 0.5, 1.0, 2.0, and 5.0 of the zeta potential ratio (*ψ_t_*/*ψ_b_*), respectively. (**a**) Shear thinning fluid (*n* = 0.8). (**b**) Shear thickening fluid (*n* = 1.2).

Figure 4 shows the dimensionless velocity (*u*/*u_s_*) distributions according to the dimensionless zeta potential difference *R* at the bottom and top for the shear thinning fluid (Figure 4a) and shear thickening fluid (Figure 4b). For comparison, the symmetric case (*R* = 0) is also included in the figure. The velocity distributions near the top and bottom walls develop from zero (on the wall) to close to the generalized Smoluchowski velocity determined by the corresponding zeta potentials; in the core region, these two velocity distributions near the walls are almost linearly connected. Therefore, as the difference in zeta potential between the top and bottom walls increases, the velocity gradient in the core region increases. The velocity gradient in the core region decreases and increases the viscosity of the shear thinning fluid and shear thickening fluid, respectively. As a result, as the dimensionless zeta potential difference *R* increases, the velocity at the center (*y*/*H* = 0) increases for shear thinning fluids and decreases for shear thickening fluids.

## 4. Conclusions

In this study, the exact solutions are proposed for fully developed two-dimensional steady unidirectional EOFs of power-law fluids with different zeta potentials at the top and bottom walls. The exact solutions are expressed in terms of Appell’s first hypergeometric functions. The effects of parameters such as the fluid behavior index *n*, electrokinetic parameter *κH*, and zeta potential *ψ_t_* and *ψ_b_* on the velocity distribution are investigated.

## Appendix A

The general integral formula for Equation (19) is

(A1) $$\begin{array}{l} \int {\left(a\sinh\left(x\right)+b\cosh\left(x\right)+c\right)}^{n}dx=\frac{1}{a\left(n+1\right)\sqrt{1-\frac{b^{2}}{a^{2}}}}\mathrm{sech}\left(\tanh^{-1}\left(\frac{b}{a}\right)+x\right)\cdot \;\\ \sqrt{\frac{a\sqrt{1-\frac{b^{2}}{a^{2}}}-i\;a\sinh\left(x\right)-i\;b\cosh\left(x\right)}{a\sqrt{1-\frac{b^{2}}{a^{2}}}+i\;c}}\sqrt{\frac{a\sqrt{1-\frac{b^{2}}{a^{2}}}+i\;a\sinh\left(x\right)-i\;b\cosh\left(x\right)}{a\sqrt{1-\frac{b^{2}}{a^{2}}}-i\;c}}\cdot {\left(a\sinh\left(x\right)+b\cosh\left(x\right)+c\right)}^{n+1}\cdot \\ F_{1}\left(1+n;\;\frac{1}{2},\;\frac{1}{2};\;2+n;\;\frac{i\left(a\sinh\left(x\right)+b\cosh\left(x\right)+c\right)}{a\sqrt{1-\frac{b^{2}}{a^{2}}}-i\;c},\frac{i\left(a\sinh\left(x\right)+b\cosh\left(x\right)+c\right)}{a\sqrt{1-\frac{b^{2}}{a^{2}}}+i\;c}\right)+const. \end{array}$$

Using Equation (A1), the primitive function of ${\left\{I\left(y\right)+C\right\}}^{\frac{1}{n}}$ in Equation (19) can be evaluated as

(A2) $$\int {\left\{I\left(y\right)+C\right\}}^{\frac{1}{n}}dy=\frac{1}{\kappa }\frac{n}{n+1}\frac{1}{\sqrt{C^{2}+w^{2}}}\frac{\left|\psi \left(y\right)\right|}{\psi \left(y\right)}{\left[I\left(y\right)+C\right]}^{\frac{n+1}{n}}\cdot \;F_{1}\left(1+\frac{1}{n};\;\frac{1}{2},\;\frac{1}{2};\;2+\frac{1}{n};\;\frac{I\left(y\right)+C}{C+iw},\frac{I\left(y\right)+C}{C-iw}\right)+const.$$

where *ψ*(*y*), *I*(*y*) and *w* are defined in Equations (10), (20), and (25), respectively. The term $\frac{\left|\psi \left(y\right)\right|}{\psi \left(y\right)}$ means the sign of the electrical potential. Since the wall zeta potentials are assumed to be negative (*ψ_t_*, *ψ_b_* < 0), *ψ*(*y*) always has a negative value; therefore, the term $\frac{\left|\psi \left(y\right)\right|}{\psi \left(y\right)}$ is −1. With all the aforementioned considerations, the velocity distribution is obtained as Equation (21).

At point *y* = *y_c_*, the two equations according to the interval in Equation (21) should have the same value.

(A3) $$u_{s}\left[V^{+}\left(y_{c}\right)-V^{+}\left(-H\right)\right]=u_{s}\left[-V^{-}\left(y_{c}\right)+V^{-}\left(H\right)\right].$$

Since $I\left(y_{c}\right)+C=0$ by Equation (18), $V^{+}\left(y_{c}\right)=V^{-}\left(y_{c}\right)=0$.

Therefore, the integral constant *C* can be obtained from the following equation

(A4) $$V^{+}\left(-H\right)+V^{-}\left(H\right)=0$$

which is simplified as Equation (27).

## Appendix B

For a symmetrical zeta potential, the *R* in Equation (17) is zero and the integral constant *C* is also zero because $\frac{du}{dy}{|}_{y=0}=0$. Owing to the symmetry, the velocity distribution can be considered only in the interval 0≤y≤H. Then, Equation (23b) becomes

(A5) $$\begin{array}{c} V^{-}\left(y\right)=\frac{1}{n+1}cosh\left(\kappa H\right){\left[\frac{\sinh\left(\kappa y\right)}{\cosh\left(\kappa H\right)}\right]}^{\frac{n+1}{n}}F_{1}\left(1+\frac{1}{n};\;\frac{1}{2},\;\frac{1}{2};\;2+\frac{1}{n};\;i\sinh\left(\kappa y\right),-\;i\sinh\left(\kappa y\right)\right)\\ =\frac{1}{n+1}sinh\left(\kappa y\right){\left[\frac{\sinh\left(\kappa y\right)}{\cosh\left(\kappa H\right)}\right]}^{\frac{1}{n}}{}_{2}F_{1}\left(\frac{1}{2},\frac{1}{2n}+\frac{1}{2};\;,\;\frac{1}{2n}+\frac{3}{2};\;-{\sin h}^{2}\left(\kappa y\right)\right) \end{array},$$

where Appell’s first hypergeometric function is reduced to the hypergeometric function _2_*F*_1_(*a*_1_, *a*_2_; *b*; *y*).

Applying Euler’s hypergeometric transformations [38,39] to the hypergeometric function in Equation (B1) gives

(A6) $$V^{-}\left(y\right)=\frac{{\left(-1\right)}^{\frac{n-1}{2n}}}{n}\frac{\cosh\left(\kappa y\right)}{\cosh^{\frac{1}{n}}\left(\kappa H\right)}{}_{2}F_{1}\left(\frac{1}{2},\frac{n-1}{2n};\frac{3}{2};\cosh^{2}\left(\kappa y\right)\right)+\frac{\sqrt{\pi }}{2n}\frac{\;\Gamma \left(\frac{n+1}{2n}\right)}{\;\Gamma \left(\frac{2n+1}{2n}\right)}\frac{{\left(-1\right)}^{\frac{n-1}{2n}}}{{\cos h}^{\frac{1}{n}}\left(\kappa H\right)}.$$

Then the velocity distribution can be obtained as

(A7) $$u\left(y\right)=u_{s}\left[-V^{-}\left(y\right)+V^{-}\left(H\right)]=u_{s}[V_{symm}\left(H\right)-V_{symm}\left(y\right)\right].$$

where *V_symm_*(*y*) is expressed in Equation (32).
